# Systematic position of the enigmatic *Mirlatia arcuata* moth resolved: a monotypic tribe within the basal branches of Larentiinae (Lepidoptera, Geometridae)

**DOI:** 10.3897/zookeys.1267.174100

**Published:** 2026-01-30

**Authors:** Pasi Sihvonen, Kyung Min Lee, Balázs Tóth, Gergely Katona, Elina Laiho, Axel Hausmann

**Affiliations:** 1 Finnish Museum of Natural History, University of Helsinki, Helsinki, Finland Hungarian National Museum Public Collection Centre Budapest Hungary; 2 Hungarian National Museum Public Collection Centre, Budapest, Hungary Finnish Museum of Natural History, University of Helsinki Helsinki Finland; 3 Bavarian State Collection of Zoology, Munich, Germany Bavarian State Collection of Zoology Munich Germany

**Keywords:** Classification, Europe, integrative taxonomy, Mirlatiini, molecular, morphology, new tribe, phylogeny

## Abstract

Recently, a new monotypic geometrid moth genus and species, *Mirlatia arcuata* Hausmann, László, Mayr & Huemer, 2023, were described from Croatia. The description was based on a single male collected in 1983, and a single female collected in 1982. The discovery of an isolated lineage in Europe was unexpected because the Geometridae fauna of the continent is likely the best explored in the world. Upon description, the systematic position of *Mirlatia arcuata* remained unresolved due to its peculiar morphology and lack of suitable molecular data to study its phylogenetic position. Based on a new specimen collected in 2024, we studied its phylogeny, including it in a maximum likelihood multi-gene global phylogenetic dataset of 1206 taxa, and comparative morphological analysis. Based on the results of our integrative approach, we propose to classify this enigmatic species in a monotypic tribe Mirlatiini Sihvonen & Hausmann, **tribe nov**. within the basal Larentiinae as the sister to Brabirodini. Other proposed taxonomic changes include reclassification of *Brabira* Moore, 1888 in Brabirodini and *Tyloptera* Christoph, 1881 in Larentiinae: *incertae sedis*. We illustrate closely related genera in Mirlatiini, Brabirodini and Dyspteridini, for taxonomic clarity.

## Introduction

The European Lepidoptera fauna is likely the best explored in the world, and the family Geometridae is no exception, thanks to a systematic revision published in the *Geometrid Moths of Europe* monographic series (Hausmann 2001; Mironov 2003; Hausmann 2004; Hausmann and Viidalepp 2012; Skou and Sihvonen 2015; Müller et al. 2019). The fauna was concluded to contain 999 species (Hausmann and Sihvonen 2019), and after the publication of the systematic checklist, a few more species have been added to the fauna of Europe and adjacent areas (e.g., Hausmann 2020; Falck and Hausmann 2020; Rajaei et al. 2021; Guerrero et al. 2021; Scalercio et al. 2021; Beshkov 2022; Werner et al. 2023). Also, phylogenetic relationships of the fauna are relatively well established (e.g., Hausmann and Sihvonen 2019; Murillo-Ramos et al. 2019; Õunap et al. 2025).

Against this background, the discovery of a distinct unknown geometrid species from Croatia was unexpected (Hausmann et al. 2023). The description was based on a single male collected in 1983 and a single female collected in 1982. The formal description highlighted the isolation, as *Mirlatia arcuata* Hausmann, László, Mayr & Huemer, 2023 was described in a monotypic genus, and its systematic position remained unresolved due to its peculiar morphology and lack of suitable molecular data. *Mirlatia arcuata* was tentatively placed in the subfamily Larentiinae based on morphological characters, including the fusion of hindwing veins Sc+R1 and Rs, and a double forewing areole supporting this. The structure of the tympanal organs, which is often diagnostic at the subfamily level, is unique: the very broad base and the lack of apical dilation of the tympanal ansa are different from any other geometrid moth. A broad ansa base is found in Archiearinae, Desmobathrinae and Ennominae: Alsophilini, but these groups have an ansa with a pointed tip (Hausmann 2001), while it is truncate in *Mirlatia*. The peculiar structures also include long bipectination in the female antennae.

**Figure 1.**
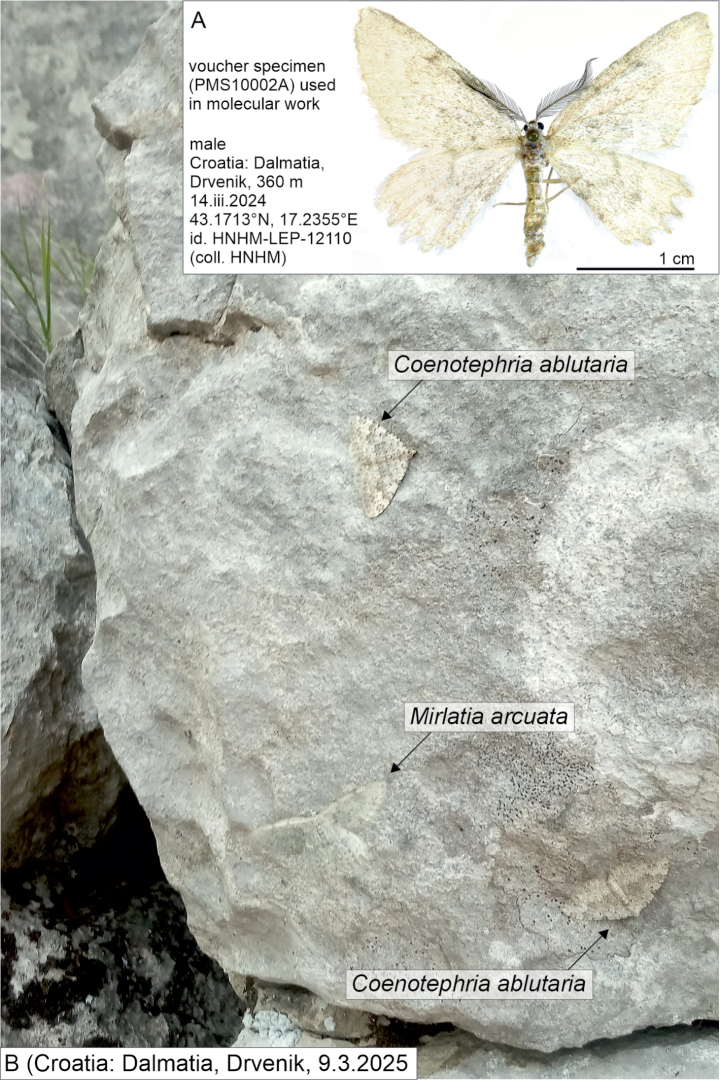
**A**. Voucher specimen of *Mirlatia arcuata*Hausmann et al. 2023 used in the molecular study; **B**. *Mirlatia arcuata* and *Coenotephria ablutaria* (Boisduval, 1840) specimens on rock, showing the strikingly cryptic coloration of both species (Croatia: Dalmatia, Drvenik, 43.1713°N, 17.2355°E, 9.3.2025). Photo 1A by Balázs Tóth, 1B by Gergely Katona.

A fresh male specimen of *Mirlatia arcuata* was found in Croatia on 14 March 2024 by Róbert Enyedi, Gergely Katona, Tamás Korompai and Balázs Tóth (Tóth et al. 2025), and again on 9 March 2025 (Fig. 1). We used tissue from the specimen collected on 14 March 2024 to analyse both mitochondrial and nuclear genes in a molecular phylogenetic dataset, which includes representatives from all continents except Antarctica; it covers all known geometrid subfamilies and the majority of tribes. We present the results of this work in this paper, in addition to the morphological examination of selected structures of *M. arcuata* and species most closely related to it as indicated by the molecular data and literature (see below), and propose a revised classification for *M. arcuata* in the Geometridae tree of life.

## Material and methods

### Abbreviations

**HNHM** Hungarian Natural History Museum, Budapest, Hungary

**NHMV** The Natural History Museum, Vienna, Austria

**NHMUK** The Natural History Museum, London, UK

**TLMF** Tiroler Landesmuseum Ferdinandeum, Innsbruck, Austria

**ZSM** SNSB - Bavarian State Collection of Zoology (Zoologische Staatssammlung München), Munich, Germany.

Label data of examined specimens are presented verbatim as they appear on the labels, a forward slash denoting separate lines and a double forward slash separating labels, and additional information about specimens or labels is enclosed in square brackets.

### Molecular analyses

Genomic DNA was extracted from a leg of the specimen collected in 2024. The procedure for DNA extraction, purification, amplification, cleaning and sequencing of both mitochondrial (COI) and protein-coding nuclear gene regions (ArgK, Wingless, RpS5, Ca-ATPase, Nex9, and EF-1alpha) (Table 1) followed the protocols described by Sihvonen et al. (2020) and Lee et al. (2024). All molecular work was conducted at the DNA laboratory of the Finnish Museum of Natural History. PCR products were sequenced at the Institute for Molecular Medicine Finland – FIMM (Helsinki, Finland). Sequence alignment, cleaning, model selection, tree search strategies using maximum likelihood (ML), node support estimation, and tree visualization also followed the aforementioned protocols.

**Table 1.** GenBank accession numbers for the new sequences used in this study.

Voucher	COI	RpS5	Wgl	EF-1alpha	Ca-ATPase	Nex9	ArgK
PMS10002A	PX412926	PX434320	PX434321	PX434318	PX434316	PX434319	PX434317

Molecular data for *Mirlatia arcuata* were analysed using a maximum likelihood approach implemented in IQ-TREE (Trifinopoulos et al. 2016), using the 1206-taxon dataset compiled by Murillo-Ramos et al. (2019). The best-fitting substitution models were selected by ModelFinder (Kalyaanamoorthy et al. 2017), with each partition assigned its own evolutionary rate. The resulting phylogenetic tree was rooted using representative species of the families Sematuridae, Epicopeiidae, Pseudobistonidae and Uraniidae. The tree was visualized and rooted in FigTree (ver. 1.4.3, Rambaut 2015) and edited for presentation in CorelDRAW (ver. 24).

### Examined material and its systematic position prior to our analyses

**Figure 2.**
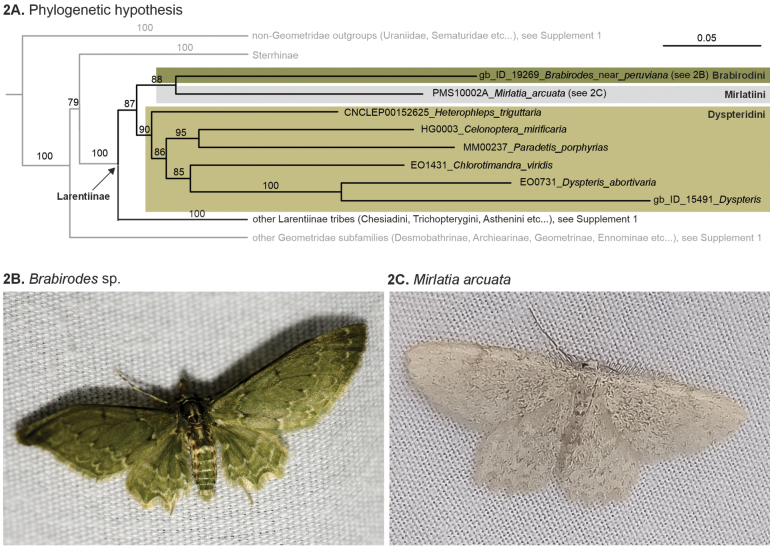
**A**. Phylogenetic position of *Mirlatia arcuata*Hausmann et al. 2023 and the monotypic Mirlatiini within the basal Larentiinae in the 1206 taxa dataset of Murillo-Ramos et al. (2019) covering Geometroidea on global scale. Only relevant branches are shown, the full tree is provided in Supplementary Material 1. The phylogenetic hypothesis is based on maximum likelihood approach of up to 11 mitochondrial and nuclear genes. Numbers above branches indicate ultrafast bootstrap support; **B**. *Brabirodes* sp. (Costa Rica: San José, 15.3.2025, photo by Jason Hafstad, used under CC-BY-NC license from iNaturalist) **C***Mirlatia arcuata* (Croatia: Drvenik, 43.1713°N, 17.2355°E, 8.3.2025, photo by Balázs Tóth).

Following the molecular analysis (Fig. 2), we examined the morphology of *Brabirodes* near peruviana Warren, 1904, which was recovered as the sister to *Mirlatia arcuata*. We also examined the morphology of *Brabira* Moore, 1888 and *Tyloptera* Christoph, 1881, which are assumed to be closely related based on the literature (Scoble 1999: Appendix; Viidalepp 2011; Beljaev 2016), and species of Dyspteridini based on literature (Hausmann and Viidalepp 2012; Hall et al. 2025). Metadata for the examined specimens, illustrated in this paper, are listed below.

#### *Mirlatia arcuata* Hausmann et al., 2023

Systematic position unclear, tentatively Larentiinae (Hausmann et al. 2023). ***Holotype male*** (DNA barcode and morphology examined): Podgora – Drvenik / YU [southern Croatia, 25 km SE Makarska] [leg. Robert] Hentscholek / [handwritten] 18.3.1983” // DNA barcode sample ID BC_ZSM_Lep_115293 // gen. prp. [genitalia preparation] Hausm. [Hausmann] G 22091 // coll. TLMF. ***Male*** (multi-gene and morphology examined): Croatia, Dalmatia, Drvenik, southern slope // ca. 360 m // light trap // 43.1713°N, 17.2355°E // 14.iii.2024 // leg. Róbert Enyedi, Gergely Katona, Tamás Korompai & Balázs Tóth; id. No HNHM-LEP-12110 // coll. HNHM. ***Paratype female*** (morphology examined): Podgora – Drvenik / YU [southern Croatia, 25 km SE Makarska] [leg. Robert] Hentscholek / [handwritten] 27.3.1982” // [gen. prp. nr. László] 3352 ♀ // coll. NHMV.

DNA barcodes available to us on BOLD https://boldsystems.org/ with the following voucher codes were examined: BC ZSM Lep 115293 (Croatia, 19.3.1983, holotype), and BC ZSM Lep 120137 (Croatia, 10.3.2024).

#### *Brabirodes* near peruviana Warren, 1904

Systematic position earlier Cidariini (Viidalepp 2011, based on *Brabirodes cerevia* (Druce, 1893)), now Brabirodini (Brehm et al. 2019). ***Male*** (morphology examined): ECUADOR/ Route Baeza – Lumbaqui / au Puente Axuela / 1530 m - 6 et 7.ii.1975 / C. Herbulot // Brabirodes / cerevia Druce / ssp. Peruviana / Warr.// Pasi Sihvonen / prep. number 2974 // coll. ZSM. ***Female*** (morphology examined): BOLIVIE Nor. Yungas / Rte. Mururata-Sta. Rosa / Rio Suapi 1200m / 1-[19]84 Lachause Porion // Pasi Sihvonen / prep. number 2975 // coll. ZSM.

DNA barcodes available to us on BOLD https://boldsystems.org/ with the following voucher codes were examined: ID 19269 (Ecuador, 5.2.2013), and MK739303 (=gb ID 19269, Ecuador, 5.2.2013).

Note: *Brabirodes* Warren, 1904 includes three taxa (Scoble 1999, Rajaei et al. 2022), which were originally validated as species (Druce 1893a, Druce 1893b, Warren 1904). In the recent literature (Rajaei et al. 2022), only two species are considered valid because taxon peruviana Warren, 1904 (type locality in Peru) is considered a subspecies of *ceravia* Druce, 1893 (type locality in Mexico). We have examined the type specimens of these three similar taxa in coll. NHMUK externally, but the type specimens are not dissected or DNA barcoded, and therefore, we cannot confirm the exact identity of our study specimens. Further, Viidalepp (2011) illustrated the male genitalia of *Brabirodes cerevia* from Nicaragua (type species of the genus), which he classified in Cidariini. Cidariini and Brabirodini are not closely related (Brehm et al. 2019), and the structures illustrated by Viidalepp (2011) are notably different from the Ecuadorian material we examined. Because the species-level taxonomy of genus *Brabirodes* is unclear, we named our study specimens as “*Brabirodes* near peruviana”.

#### *Brabira atkinsoni* Moore, 1888

Systematic position Dyspteridini (Beljaev 2016). ***Male*** (morphology examined): NEPAL / 18 Km SSE Katmandu / Route du Phulchoki / 2100 m, 1.X.1983 / C. Herbulot // Pasi Sihvonen / prep. number 2976 // coll. ZSM. ***Female*** (morphology examined): Nepal / Prov. Nr. 1 East / Pultschuk 23.–2500m / 12.VI.1967 leg. / Dierl-Forster-Schacht / Staatsslg. München // Pasi Sihvonen / prep. number 2977 // coll. ZSM.

DNA barcodes available to us on BOLD https://boldsystems.org/ with the following voucher codes were examined: BC ZSM Lep 29780 (China: Sichuan, 17.7.2009), BC ZSM Lep 29989 (China: Sichuan, 13.7.2009), BC ZSM Lep 29992 (China: Sichuan, 13.7.2009), Brabira AH01Ch (China: Sichuan, 13.7.2009), BC ZSM Lep 38274 (Bhutan, 25.10.2009), BC ZSM Lep 68687 (Nepal, 15.7.1995). These belong to three different BINs: BOLD:AAJ8062, BOLD:AAP1598, BOLD:ACL9748.

#### *Tyloptera bella* (Butler, 1878)

Systematic position Dyspteridini (Viidalepp 2016). ***Male*** (morphology examined): Russia S.B. / Vladivostok dist. 20 / Nachodka / 07.[19]94. / leg. Kuznezov // Pasi Sihvonen / prep. number 2978 // coll. ZSM. ***Female*** (morphology examined): [RUSSIA] Vladivostok / Siberie Or. / 2-VIII-1925 // Pasi Sihvonen / prep. number 2979 // coll. ZSM.

DNA barcodes available to us on BOLD https://boldsystems.org/ with the following voucher codes were examined: AYK-04-1013-13 (Japan, 1.8.2004), AYK-06-7336 (Japan, 26.8.2006), 09-JDWGEO-095 (Japan, 26.8.2006), BC ZSM Lep 29687 (China: Sichuan, 12.7.2009), BC ZSM Lep 29945 (China: Sichuan, 12.7.2009), BC ZSM Lep 68691 (Russia: Vladivostok, 31.7.1994). These belong to two different BINs: BOLD:AAF0396, BOLD:AAI9155.

### Morphological analyses

Adult specimens, genitalia, and abdomens were prepared and photographed following methods summarized in Sihvonen et al. (2020). Uneverted vesica and tympanal organs were photographed in situ during dissection to allow an optimal angle for observation and illustration.

One pair of wings was removed from the dry specimen and submerged in 99% ethanol for a few seconds, and then submerged in 2.7% sodium hypochlorite (NaClO) solution for a few minutes for bleaching. Following this, the wings were submerged in 99% ethanol to remove NaClO and to physically remove the remaining scales with delicate brushes. The wing slides were left unstained and mounted in Euparal.

Photographs were edited in Adobe Photoshop (ver. CS6), and figure plates were compiled in CorelDRAW (ver. 24).

## Results

### Biology

Specimens caught in 2024 and 2025 were attracted to portable UV light tubes, placed in forest patches with *Ostrya carpinifoloa* Scop. and herbaceous vegetation with *Erica* spp. right at the edge of a scree at about 360 meters. Potential habitats of *M. arcuata* include steep slopes, cliffs and screes. The collecting locality and vegetation are described in more detail in Tóth et al. (2025). The co-occurring moth fauna included overwintering species, typical of late winter and early and mid-spring.

*Mirlatia arcuata* has a planiform resting position, i.e., the wings are parallel to the ground, and it camouflages itself against the rocks (Figs 1, 2).

### Phylogenetic analysis

Inclusion of *Mirlatia arcuata* in a global, multi-gene molecular analysis of Geometroidea dataset of 1206 terminal taxa placed the species within the basal Larentiinae, as sister to Brabirodini (ultrafast bootstrap support = 88) (Fig. 2). Genera *Brabirodes* + *Mirlatia* were recovered as sister to Dyspteridini (ultrafast bootstrap support = 87). The full phylogeny is provided in Suppl. material 1.

### Morphology

Comparative morphological analysis revealed the uniqueness of *Mirlatia arcuata* (Figs 7–10, 11–14, 15–18). When compared to *Brabirodes* near peruviana, *Brabira atkinsoni* and *Tyloptera bella*, or more widely to Dyspteridini (Viidalepp 2011; Hausmann and Viidalepp 2012; Hall et al. 2025), autapomorphic characters in the male genitalia include a bilobed uncus, densely spinose juxta and a bundle of cornuti. In the female genitalia, the diagnostic characters (when compared to other basal Larentiinae) include anterior apophyses, which are interconnected by a narrow, strongly sclerotized, evenly-arched band, and a corpus bursae with a striated posterior part. Further, the apex of the ansa in the tympanal organ is not expanded laterally.

The examined basal Larentiinae taxa share characters, which are potential synapomorphies waiting to be tested in an analytical context: forewing with two areoles (*Mirlatia + Brabirodes + Brabira*) and the narrow sclerotized, evenly-arched band between anterior apophyses (*Mirlatia + Brabirodes*).

*Brabirodes* and *Brabira* are the most similar among the examined taxa, sharing, for instance, greenish wings, reduced hindwings with crenulate margins, an anal margin of the hindwing with a lobe, reduced venation on the hindwing, two forewing areoles and long labial palps. Further structural similarities include, for instance, in the male genitalia a large vesica, in which the ductus ejaculatorius opens laterally near the base and is basally narrow and restricted (Figs 8, 9).

*Tyloptera bella* is morphologically different from the other examined genera: the forewing has only one areole, the hindwing has three M veins, the gnathos arms are fused, the ductus ejaculatorius does not open laterally, the apex of the ansa is expanded laterally and “frilled” (margins are not smooth), and the margin of the membrane in the tympanal organ is crown-shaped (Figs 10, 14, 18).

### Classification

Integrative data based on molecules and morphology (Figs 2, 3–6, 7–10, 11–14, 15–18, 19–20, 21–22) support the view that *Mirlatia* is a monotypic tribe, morphologically distinct from the sister lineage Brabirodini: *Brabirodes* + *Brabira*. We classify *Mirlatia* here in a monotypic tribe Mirlatiini Sihvonen & Hausmann, tribe nov. (Table 2). Among the European Geometridae, we place Mirlatiini: *Mirlatia arcuata* in the linear list of taxa (Hausmann and Sihvonen 2019; the phylogenetic relationships are refined in Õunap et al. (2025) before *Celonoptera mirificaria* Lederer, 1862, which is currently classified in Dyspteridini (Õunap et al. 2025).

**Figures 3–6.**
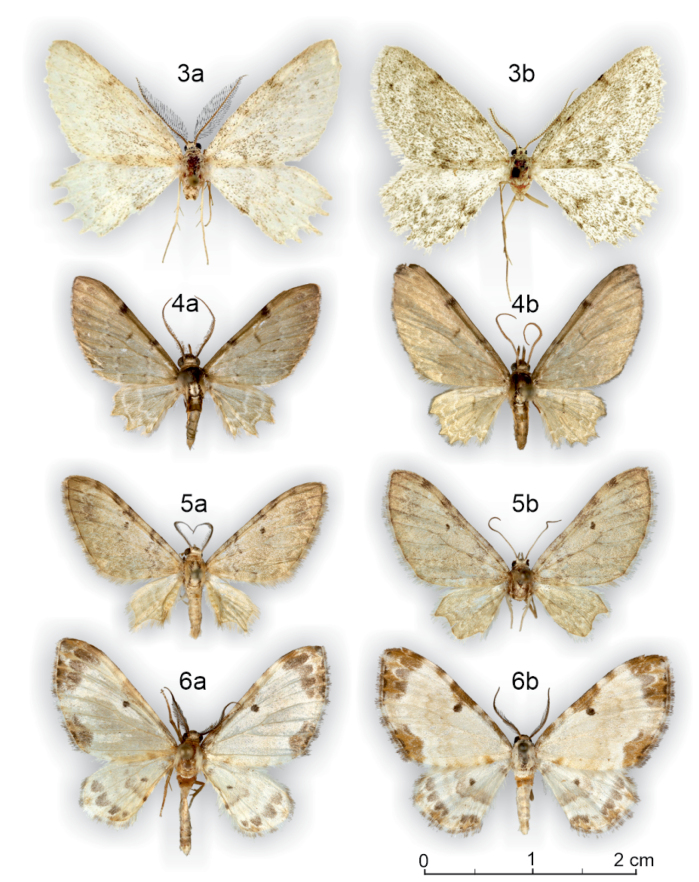
Adults of the examined species, with specimen metadata below. **3a**. *Mirlatia arcuata* ♂ (holotype), Croatia: Podgora - Drvenik, 18.3.1983 (coll. TLMF). Barcoded (BC_ZSM_Lep_15293), dissected (Hausmann G 22091); **3b**. *M. arcuata* ♀ (paratype), Croatia: Pogdora - Drvenik, 27.3.1982 (coll. NHMV). Dissected (László 3352). **4a**. *Brabirodes* near peruviana ♂, Ecuador: Puente Azuela, 6–7.2.1975 (coll. ZSM). Dissected (Sihvonen PMS2974); **4b**. *B.* near peruviana ♀, Bolivia: Rio Suapi, 1200 m, January 1984 (coll. ZSM). Dissected (Sihvonen PMS2975). **5a**. *Brabira atkinsoni* ♂, Nepal: 18 km SSE Kathmandu, 2100 m, 1.10.1983 (coll. ZSM). Dissected (Sihvonen PMS2976); **5b**. *B. atkinsoni* ♀, Nepal: East Pultschuk, 2500 m, 12.6.1967 (coll. ZSM). Dissected (Sihvonen PMS2977). **6a**. *Tyloptera bella* ♂, Russia: Vladivostok, Nakhodka, July 1994 (coll. ZSM). Dissected (Sihvonen PMS2978); **6b**. *T. bella* ♀, Russia: Vladivostok, 2.8.1925 (coll. ZSM). Dissected (Sihvonen PMS2979).

**Figures 7–10.**
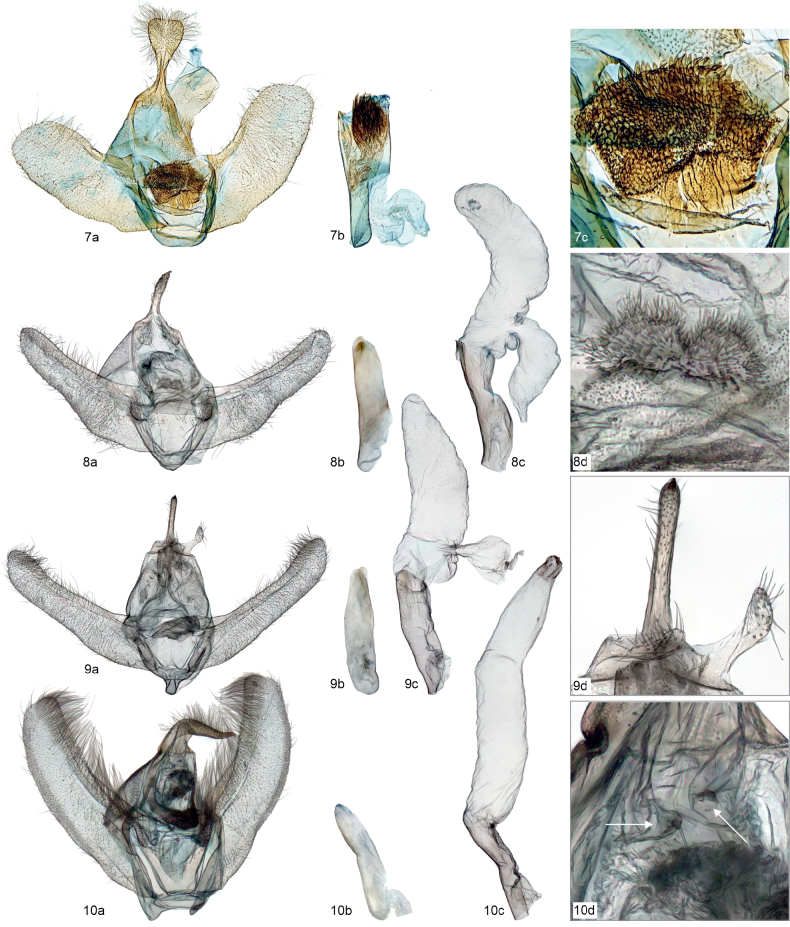
Male genitalia (not to scale) of the examined species, with specimen metadata below. **7**. *Mirlatia arcuata*. **7a**. Genitalia; **7b**. Aedeagus; **7c**. Еnlarged juxta. Holotype, Croatia: Podgora - Drvenik, 18.3.1983 (coll. TLMF, dissection Hausmann G 22091). **8**. *Brabirodes* near peruviana. **8a**. Genitalia; **8b**. Aedeagus; **8c**. Vesica; **8d**. Enlarged spinose structure. Ecuador: Puente Azuela, 6–7.2.1975 (coll. ZSM, dissection Sihvonen PMS2974). **9**. *Brabira atkinsoni*. **9a**. Genitalia; **9b**. Aedeagus; **9c**. Vesica; **9d**. Enlarged juxta and socii. Nepal: 18 km SSE Kathmandu, 2100 m, 1.10.1983 (coll. ZSM, dissection Sihvonen PMS2976). **10**. *Tyloptera bella*. **10a**. Genitalia; **10b**. Aedeagus; **10c**. Vesica; **10d**. Enlarged socii. Russia: Vladivostok, Nakhodka, July 1994 (coll. ZSM, dissection Sihvonen PMS2978).

**Figures 11–14.**
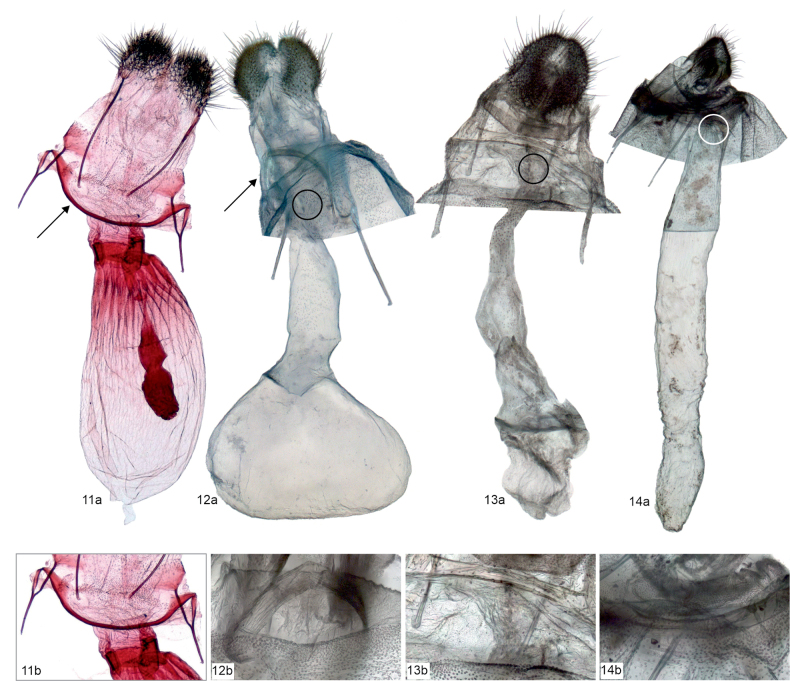
Female genitalia (not to scale) of the examined species, with specimen metadata below. **11**. *Mirlatia arcuata*. **11a**. Genitalia; **11b**. Ostium bursae and adjacent structures. Croatia: Pogdora - Drvenik, 27.3.1982 (coll. NHMV, dissection László 3352). **12**. *Brabirodes* near peruviana. **12a**. Genitalia; **12b**. Ostium bursae and adjacent structures. Bolivia: Rio Suapi, 1200 m, January 1984 (coll. ZSM, dissection Sihvonen PMS2975). **13**. *Brabira atkinsoni*. **13a**. Genitalia; **13b**. Ostium bursae and adjacent structures. Nepal: East Pultschuk, 2500 m, 12.6.1967 (coll. ZSM, dissection Sihvonen PMS2977). **14**. *Tyloptera bella*. **14a**. Genitalia; **14b**. Ostium bursae and adjacent structures. Russia: Vladivostok, 2.8.1925 (coll. ZSM, dissection Sihvonen PMS2979).

**Figures 15–18.**
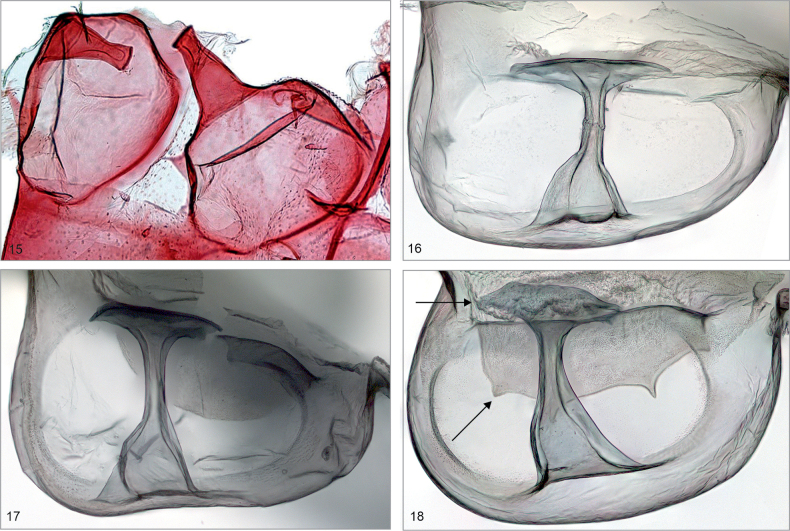
Tympanal organs (not to scale) of the examined species, with specimen metadata below. **15**. *Mirlatia arcuata*, female. Croatia: Pogdora - Drvenik, 27.3.1982 (coll. NHMV, dissection László 3352). **16**. *Brabirodes* near peruviana, female. Bolivia: Rio Suapi, 1200 m, January 1984 (coll. ZSM, dissection Sihvonen PMS2975). **17**. *Brabira atkinsoni*, female. Nepal: East Pultschuk, 2500 m, 12.6.1967 (coll. ZSM, dissection Sihvonen PMS2977). **18**. *Tyloptera bella*, female. Russia: Vladivostok, 2.8.1925 (coll. ZSM, dissection Sihvonen PMS2979).

**Figures 19, 20.**
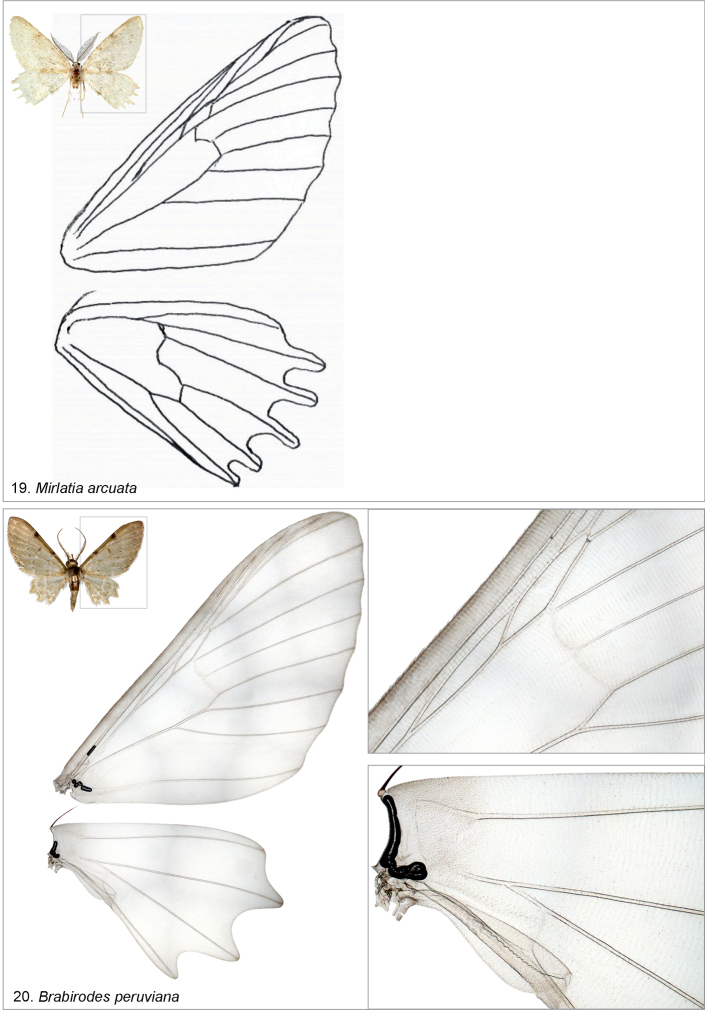
Wing venations (not to scale) of the examined species, with specimen metadata below. **19**. *Mirlatia arcuata*, holotype male. Croatia: Podgora-Drvenik, 18.3.1983 (coll. TLMF, gen. prep. Hausm. G 22091). **20**. *Brabirodes* near peruviana, male. Ecuador: Puente Azuela, 6–7.2.1975 (coll. ZSM, dissection PMS2980).

**Figures 21, 22.**
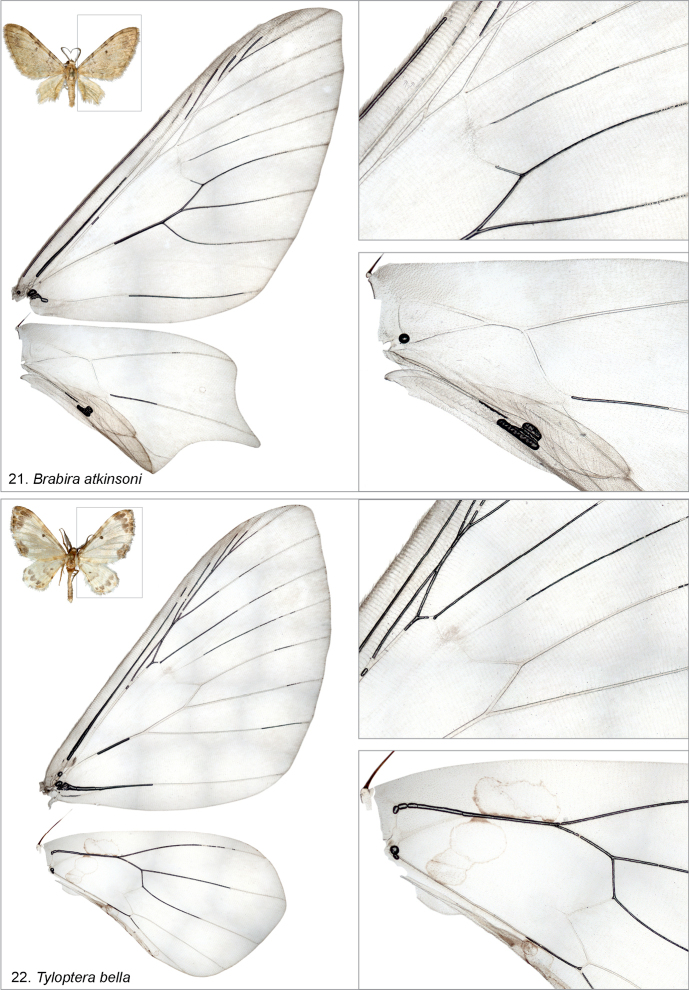
Wing venations (not to scale) of the examined species, with specimen metadata below. **21**. *Brabira atkinsoni*, male. Nepal: 18 kkm SSE Kathmandu, 1.10.1983 (coll. ZSM, dissection PMS2981). **22**. *Tyloptera bella*, male. Russia: Vladivostok, July 1994 (coll. ZSM, dissection PMS2982).

**Table 2.** Summary of formally proposed taxonomic changes and diagnostic morphological characters of the basal Larentiinae. Taxonomic changes are arranged alphabetically in “Explanation” column. Diagnostic morphological characters are based on the literature and on the present study. Most of the listed characters are unambiguous at the tribe level and show exceptions, but when considered together, they allow delimitation of tribes as monophyletic groups. The classification of all listed genera in the tribes has not been tested analytically; therefore it is tentative. Species diversity derived from Rajaei et al. (2022); biogeographic realms after Olson et al. (2001).

Explanation		Taxonomic change
*Brabira* Moore, 1888 is transferred from Dyspteridini (Beljaev 2016) to Brabirodini.		reclassification to Mirlatiini tribe nov.
We propose Mirlatiini Sihvonen & Hausmann as a valid tribe, based on *Mirlatia arcuata*Hausmann et al. 2023. The diagnostic characters of *Mirlatia* also apply at the tribe level (Hausmann et al. 2023).		tribe nov.
*Tyloptera* Christoph, 1881 is transferred from Dyspteridini (Beljaev 2016) to Larentiinae: *incertae sedis*. Tribe classification awaits further studies.		tribe combination *incertae sedis*.
Tribe	Diagnostic (synapomorphic) characters	Diversity and distribution
Mirlatiini	Uncus dilated, spoon-shaped; apex of ansa not dilated; juxta rugose, spinulose; vesica with group of cornuti; base of abdomen not specialized; hindwing margin crenulate; forewing with two areoles; female antennae bipectinate; papillae anales connected by strongly sclerotized ridge (Hausmann et al. 2023, present study).	1 genus, 1 species: *Mirlatia arcuata*. West Palearctic (Europe)
Brabirodini	Wings dominantly greenish; hindwings reduced; hindwing margin crenulate; forewing with two areoles; apex of ansa dilated, wide; juxta membranous; cornuti absent; ductus ejaculatorius opens laterally; base of abdomen not specialized; female antennae filiform (present study).	2 genera, 12 species: *Brabirodes* 3, *Brabira*, 9. Neotropical, Indo-Malay, East Palearctic
Dyspteridini	Base of abdomen with long, setose apodeme; hollow sac on A2 absent; wings dominantly green; hindwing margin smooth; vesica with bundle of small cornuti (Viidalepp 2011). Apex of ansa dilated, wide; hindwings reduced; forewings with two areoles; female antennae filiform (present study).	5 genera, 40 species: *Heterophleps* 36, *Celonoptera* 1, *Paradetis* 1, *Chlorotimandra* 1, *Dyspteris* 1. Indo-Malay, Nearctic, Palearctic, Australasia, Neotropical

*Brabira* has recently been classified in Trichopterygini (Holloway 1997, Viidalepp 2011) or in Dyspteridini (Beljaev 2016). Here we demonstrate that at least the type species of *Brabira* (*B. atkinsoni* Moore, 1888) is morphologically similar to the type species of Brabirodini: *Brabirodes* (*B. peruviana* Warren, 1904). The similarity concerns external characters such as elongated labial palps, wing color and pattern and shape, and wing venation. In the male genitalia, these two genera share, for instance, the vesica shape with a laterally opening ductus ejaculatorius (Figs 4, 5, 8, 9, Table 2). The close connection of these genera was noted already by Warren (1904). Holloway (1997) illustrates *Brabira emerita* Prout, 1926 from Borneo, in which the corpus bursae of the female genitalia is similarly striated to that of *Mirlatia arcuata*. We transfer *Brabira* Moore, 1888 to Brabirodini new classification.

Based on the morphological data, we note that *Tyloptera bella*, which has been classified earlier in Trichopterygini (Viidalepp 2011) and Dyspteridini (Beljaev 2016), does not have diagnostic characters of either of these tribes, nor Brabirodini or Mirlatiini (Table 2). The tympanal organ is unique, having a membrane with a spiked margin, and the apex of the ansa is membrane-like, frilled, and wide. The authors have not seen such a spiked margin (crown-shaped) elsewhere in Geometridae. *Tyloptera bella* also has one areole on the forewing (other examined Larentiinae have two), the inner margin of the hindwing is without a lobe, it has a well-developed uncus, paired socii, and fused gnathos arms. The closest non-congeneric DNA barcode matches are *Afroracotis lydiae* László et al. 2023 (Ennominae: Boarmiini, 5.56%; due to long-branch attraction) and the closest Larentiinae is *Heterophleps triguttaria* Herrich-Schäffer, 1854 (Dyspteridini, 6.38%). *Tyloptera bella* has not yet been included in a multi-gene molecular phylogeny. We classify it as Larentiinae: *incertae sedis*, noting that further research is needed to clarify its systematic position.

## Discussion

In this paper, we examined the phylogeny of the monotypic *Mirlatia arcuata* from Croatia and provided evidence that it belongs to the basal evolutionary lineage within the subfamily Larentiinae. In general, understanding phylogeny is significant because it depicts the evolutionary relationships between organisms, thereby providing a framework for understanding how life has diversified. This knowledge can be used, for instance, to study the evolutionary history of species and the evolution of traits over time, and it can be applied, for instance, in nature conservation by identifying isolated lineages. *Mirlatia arcuata* is a case in point: it is a phylogenetically isolated evolutionary lineage and therefore of potential conservation value, which we highlight as a priority for further research. Its biology, habitat and life history need to be investigated; the host plant of the caterpillar needs to be identified; its phenology must be understood; and its distribution needs to be mapped. These are prerequisites for making knowledge-based decisions about its conservation.

Our study also demonstrates that an integrative approach is needed to understand how an isolated lineage in the phylogenetic tree should be classified. If the molecular phylogenetic hypothesis is used alone, we cannot infer from the tree whether *Mirlatia arcuata* and its sister lineage should be classified as a single taxonomic entity. Morphology shows clearly that the sister lineages are structurally different, and therefore it is logical to classify them in different taxonomic entities, in this case different tribes.

The basal Larentiinae have interesting biogeographic patterns. In the molecular analysis, the West-Palearctic *Mirlatia arcuata* was recovered as sister to the Neotropical *Brabirodes* near peruviana. Our morphological analysis, however, shows that *Brabirodes* is closely related to the East Palearctic *Brabira*; therefore, *M. arcuata* has connections to both the East Palearctic and Neotropical faunas. The connection between Palearctic and Neotropical biogeographical realms is rare in geometrid moths, but a somewhat similar cross-Atlantic connection was identified recently for a geometrid moth in the Canary Islands (Sihvonen et al. 2025) and earlier for the predominantly Palaearctic genus *Rhodostrophia* with three, clearly congeneric species in Chile (Trusch and Hausmann 2007). We note that the current taxon sampling in our multi-gene molecular phylogeny remains limited, and the addition of new taxa could potentially change the relationships.

Finally, we note that the socii are present in all examined species (Figs 7–10, including enlarged photos) and the gnathos is present in *Tyloptera bella* (Fig. 10). Traditionally, Larentiinae have been considered to lack a gnathos (Agnathoi group sensu Pierce 1914), and this view has been repeated in recent literature (see Schmidt 2015 for review and references). Similarly, many publications have assumed that the socii are generally lacking in Larentiinae (Schmidt 2017). Our results support the views of Schmidt (2015, 2017) that both structures are widely present in Larentiinae. Maybe the socii have been overlooked previously because of their small size, but the presence of small and setose lobes at the base of the uncus are diagnostic (Figs 9d, 10d).

## Additional information

### Use of AI

No use of AI was reported.

### Author ORCIDs

Pasi Sihvonen https://orcid.org/0000-0003-2237-9325

Kyung Min Lee https://orcid.org/0000-0001-9055-1073

Balázs Tóth https://orcid.org/0000-0002-6089-1218

Gergely Katona https://orcid.org/0000-0002-3161-8060

Elina Laiho https://orcid.org/0009-0004-6497-4178

Axel Hausmann https://orcid.org/0000-0002-0358-9928

### Data availability

All of the data that support the findings of this study are available in the main text or Supplementary Information.

## Supplementary materials

Supplementary material 1 Molecular maximum likelihood phylogenetic hypothesis, including *Mirlatia arcuata* (highlighted in orange), inference by gene, full tree of 1206 terminal taxa Data type pdf Explanation note The analysis includes up to seven genes per terminal taxa. Numbers under the branches are support values SH-aLRT support (%) / ultrafast bootstrap support (%) from IQTREE2. A small part of the tree is shown in Fig. 2. https://binary.pensoft.net/file/1522886This dataset is made available under the Open Database License (http://opendatacommons.org/licenses/odbl/1.0/). The Open Database License (ODbL) is a license agreement intended to allow users to freely share, modify, and use this Dataset while maintaining this same freedom for others, provided that the original source and author(s) are credited. Pasi Sihvonen, Kyung Min Lee, Balázs Tóth, Gergely Katona, Elina Laiho, Axel Hausmann
