# Supplementary figures and images for: Systematic position of the enigmatic Mirlatia arcuata moth resolved: a monotypic tribe within the basal branches of Larentiinae (Lepidoptera, Geometridae)
Source: Zookeys. 2026 Jan 30;1267:355–72. doi: 10.3897/zookeys.1267.174100 (PMC12881918; doi:10.3897/zookeys.1267.174100)

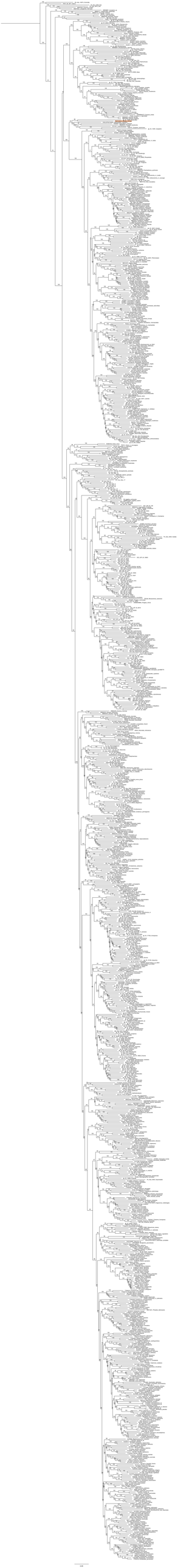

Supplement: Supplementary material 1 — Molecular maximum likelihood phylogenetic hypothesis, including Mirlatia arcuata (highlighted in orange), inference by gene, full tree of 1206 terminal taxa [file zookeys-1267-355_article-174100__-s001.pdf]
